# Focal hyperechoic liver lesions in children: far beyond hemangiomas - pictorial essay

**DOI:** 10.1590/0100-3984.2018.0119

**Published:** 2020

**Authors:** Silvia Maria Sucena da Rocha, Maurício Gustavo Ieiri Yamanari, Marcia Wang Matsuoka, Gisele Correa Almeida, Flavia Aiko Sakamoto, Lisa Suzuki

**Affiliations:** 1 Instituto da Criança do Hospital das Clínicas da Faculdade de Medicina da Universidade de São Paulo (ICr/HC-FMUSP), São Paulo, SP, Brazil.; 2 Centro Diagnóstico do Laboratório Fleury Medicina e Saúde, São Paulo, SP, Brazil.; 3 Hospital Israelita Albert Einstein, São Paulo, SP, Brazil.; 4 Centro de Diagnósticos do Hospital Infantil Sabará, São Paulo, SP, Brazil.

**Keywords:** Liver/diagnostic imaging, Ultrasonography, Liver neoplasms/diagnosis, Diagnosis, differential, Infant, Child, Adolescent, Fígado/diagnóstico por imagem, Ultrassonografia, Neoplasias hepáticas/diagnóstico, Diagnóstico diferencial, Lactente, Criança, Adolescente

## Abstract

The aim of this report was to present a selection of focal hyperechoic liver lesions of different etiologies, illustrating the wide spectrum of diagnostic possibilities for such lesions in the pediatric population.

## Abstract (pt)

Apresentamos uma seleção de lesões focais hiperecogênicas hepáticas de diversas etiologias, que ilustram o grande espectro de diferentes possibilidades diagnósticas dessas lesões na faixa pediátrica, muito além dos hemangiomas.

## INTRODUCTION

Focal hyperechoic liver lesions in children encompass a wide range of diagnostic possibilities including congenital and acquired lesions, neoplastic and non-neoplastic lesions, benign and malignant lesions. Knowledge of the spectrum of such lesions, their imaging aspects, the age group in which they are most prevalent, their clinical characteristics, the associated laboratory test results, and the patient history (of trauma or intervention) is essential for the diagnosis.

Abdominal ultrasound images were obtained at our institution. We selected cases of the following: childhood hepatic hemangioma, congenital hepatic arteriovenous malformation, umbilical vein catheterization hematoma, hepatoblastoma, focal steatosis, hepatic lipoma, biliary rhabdomyosarcoma, regeneration nodule and biliary hamartomas.

## INFANTILE HEPATIC HEMANGIOMA OR HEMANGIOENDOTHELIOMA

The most common benign childhood liver tumor of vascular origin is infantile hemangioma, which is typically characterized by a growth phase followed by a regression phase. Approximately 90% of cases are diagnosed in the first six months of life, and it is slightly more common in girls.

Although most childhood hemangiomas manifest as an asymptomatic abdominal mass, life-threatening complications can occur: high-output congestive heart failure, due to intratumoral arteriovenous shunts; the coagulopathy known as Kasabach-Merritt syndrome, as a consequence of platelet sequestration; and severe hypothyroidism, caused by the activity of type 3 iodothyronine deiodinase, which is produced at high levels by the tumor. Laboratory tests reveal anemia and hyperbilirubinemia. Serum α-fetoprotein levels are rarely elevated.

**Figure 1:**
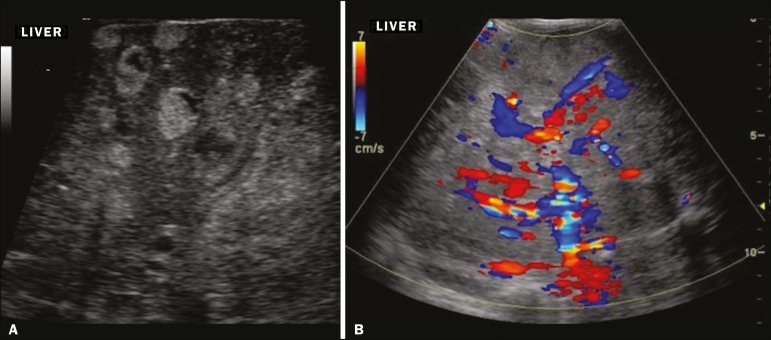
Childhood hepatic hemangioma-diffuse lesions in a 6-month-old girl. A: B-mode ultrasound shows multiple diffusely distributed hyperechoic liver nodules, some with small cystic areas. B: Color Doppler study showing a diffuse increase in hepatic vascularization.

**Presentation on ultrasound** - Solitary, multifocal, or diffuse lesions (Figure 1), which determine the imaging characteristics: large focal lesions are often heterogeneous, with cystic areas and calcifications; multifocal lesions are small, usually hypoechoic, and uniform in appearance; in diffuse disease, the liver is massively enlarged and replaced by multiple, usually hyperechoic, masses with a mass effect on the adjacent organs. Evidence of high flow (enlargement of the hepatic arteries and veins, together with thinning of the aorta below the origin of the celiac trunk) can be found in large solitary lesions and in diffuse disease^(1)^.

## CONGENITAL HEPATIC ARTERIOVENOUS MALFORMATION

**Figure 2:**
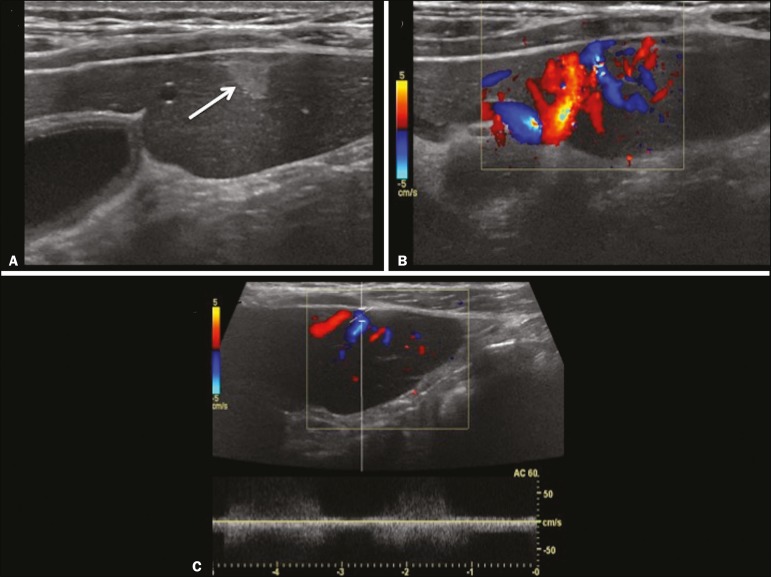
Congenital hepatic arteriovenous malformation in a 4-year-old girl with hereditary hemorrhagic telangiectasia who presented with complex hepatic vascular malformation. A: B-mode ultrasound shows subcapsular hyperechoic focal lesion (telangiectasia). B: Doppler ultrasound showing tortuous large vessels towards the lesion. C: Spectral ultrasound analysis showing turbulent flow at high velocity. Signs of portal hypertension, such as reversal of the portal venous flow and collateral circulation, were present at diagnosis (images not included).

Congenital hepatic arteriovenous malformations are vascular abnormalities in which there is direct communication between the arterial and venous systems without neoplastic tissue between them (Figure 2). They are present from birth and have no potential for growth or involution. Typically, there is hyperdynamic circulation due to arteriovenous or portal anastomoses, which can lead to high-output heart failure. Other complications include extrahepatic shunts, portal hypertension, anemia, and hepatomegaly.

Arterioportal fistulas can be associated with hereditary hemorrhagic telangiectasia (Osler-Weber-Rendu syndrome), Ehlers-Danlos syndrome, and biliary atresia^(2)^.

**Presentation on spectral and color Doppler ultrasound** - Faint hyperechoic areas containing tortuous, dilated vessels with a pattern of low-resistance arterial flow and pulsatile venous flow, together with signs of portal hypertension.

## POST-CATHETERIZATION HEMATOMA OF THE UMBILICAL VEIN

**Figure 3:**
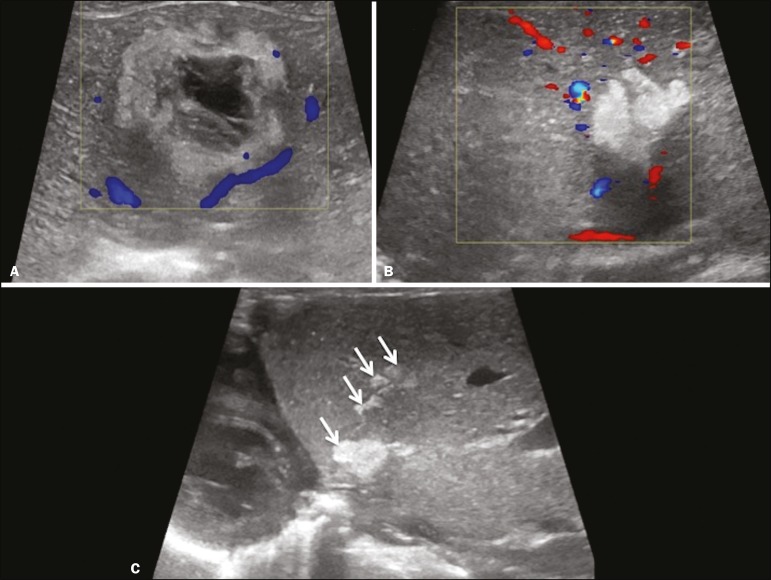
Hematoma as a complication of misplaced umbilical catheter. Ultrasound examinations of three different newborns, at two, four, and six weeks of age, respectively, showing hyperechoic focal lesions with irregular borders. In A, there is a liquid component in the center of the lesion. In C, the lesion is elongated (arrows), probably representing the path of the catheter that transfixed the hepatic parenchyma.

Hepatic complications of poor positioning of an umbilical venous catheter are uncommon and occur due to extravasation of blood, drugs, or parenteral nutrition into the liver tissue. Hepatic complications include hematoma (Figure 3), fluid collections, hepatic vessel thrombosis, and hepatic necrosis. The differential diagnoses include liver abscess, hamartoma, hemangioma, and hepatoblastoma^(3,4)^.

## HEPATOBLASTOMA

A hepatoblastoma is a malignant neoplasm whose cells resemble those of the embryonic liver and is the most common primary liver tumor in children. In 68% of cases manifest in the first two years of life and 90% are observed in patients under five years of age, with predominance in boys. A typical feature of this tumor is a highly elevated serum α-fetoprotein level.

**Figure 4:**
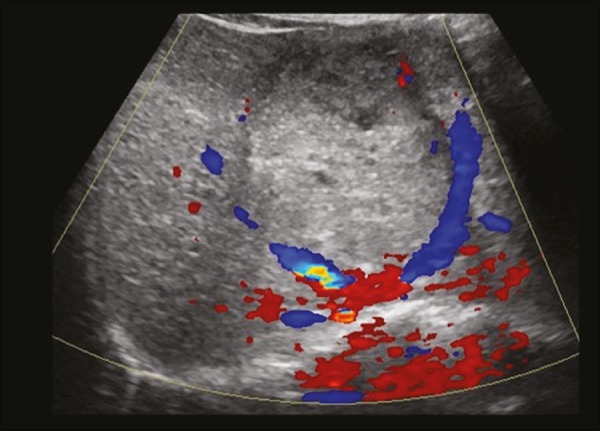
Hepatoblastoma in a 4-year-old girl. Doppler ultrasound showing a heterogeneous, slightly hyperechoic mass, containing tiny cystic foci, that displaces the hepatic veins. Pathology result of the surgical specimen obtained by resection of the hepatoblastoma.

**Presentation on ultrasound** - Circumscribed liver mass with lobulated contours (Figure 4), whose appearance depends on the histological type. Hepatoblastomas are mostly hyperechoic, being homogeneous in cases of epithelial hepatoblastomas and heterogeneous in cases of mixed tumors, with calcifications and cystic areas of hemorrhage/necrosis^(5)^.

## FOCAL STEATOSIS

**Figure 5:**
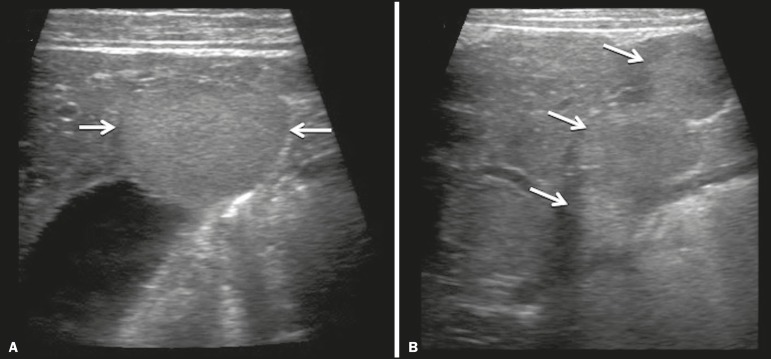
Multifocal nodular subcapsular hepatic steatosis in an 8-year-old boy with chronic liver disease and cystic fibrosis. Follow-up ultrasound showing diffuse heterogeneity of the liver parenchyma and subcapsular hyperechoic multifocal lesions, some with a nodular appearance (arrows), without causing a mass effect.

The most common pattern of hepatic steatosis is diffuse homogeneous fat deposition. Less common patterns of deposition include focal (adjacent to the falciform ligament, in the porta hepatis, within the biliary fossa), diffuse heterogeneous, and multifocal/nodular (Figure 5). The multifocal pattern is uncommon in childhood and is characterized by multiple foci of fat accumulation scattered at atypical locations throughout the liver.

**Presentation on ultrasound** - Hyperechoic lesions, which do not promote a mass effect or displace branches of the hepatic and portal veins, which cross the lesions without altering their course. Lesions may mimic true nodules, causing diagnostic difficulty, especially in the event of pre-existing chronic liver disease or malignancy^(6,7)^.

## HEPATIC LIPOMA

A hepatic lipoma is a quite uncommon, benign lesion that is usually asymptomatic. Although lipomas may consist only of fat cells, they may also contain tissue that is adenomatous (adenolipoma), angiomatous (angiomyolipoma) or myomatous (myelolipoma). They are occasionally accompanied by tuberous sclerosis (in 6% of cases) or renal angiomyolipoma (in 20%).

**Figure 6:**
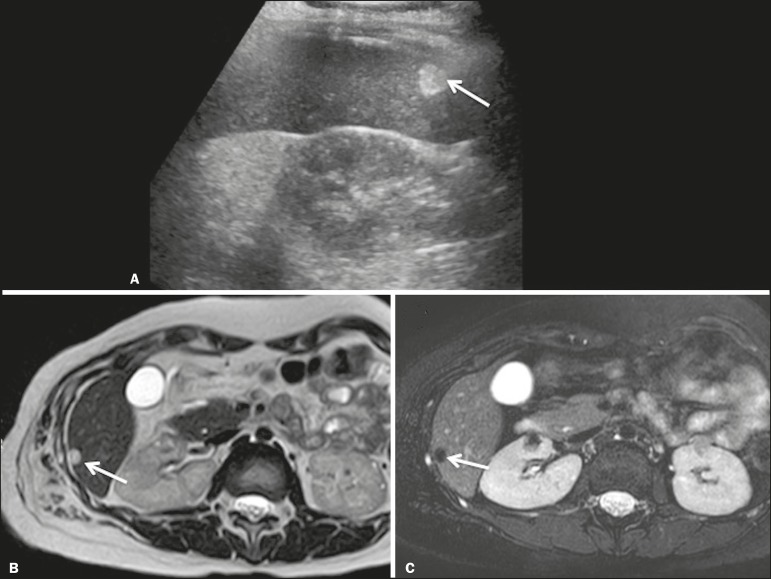
Hepatic lipoma in a 15-year-old female with tuberous sclerosis. A: Ultrasound of the liver, showing a hyperechoic circumscribed nodular lesion (arrow). B: T2-weighted magnetic resonance imaging sequence showing a small, round lesion with a clearly hyperintense signal (arrow) and evident signal loss following fat suppression (arrow in C), behavior similar to that of subcutaneous tissue, proving that the lesion had a fat component and confirming the diagnosis of lipoma.

**Presentation on ultrasound** - Well circumscribed, uniformly hyperechoic lesion with posterior acoustic shadowing of varying intensity^(7)^, as shown in Figure 6.

## RHABDOMYOSARCOMA OF THE BILIARY TRACT

A rhabdomyosarcoma is a highly aggressive tumor that can occur anywhere in the body and rarely appears in the biliary tree^(5)^. Biliary rhabdomyosarcoma occurs almost exclusively in children and accounts for 1% of liver tumors in the pediatric population. In 75% of cases, they are diagnosed before the age of five. The clinical manifestations are jaundice, bloating, fever, hepatomegaly, nausea, and vomiting. Laboratory tests reveal elevated levels of conjugated bilirubin and alkaline phosphatase, together with normal serum α-fetoprotein levels.

Rhabdomyosarcomas are usually large. Polypoid, or “bunch-of-grapes”, projections are often observed in the lumen of the bile duct. Although the extrahepatic bile ducts are the most commonly involved, the lesion can originate or grow in the intrahepatic bile ducts and invade the liver.

**Figure 7:**
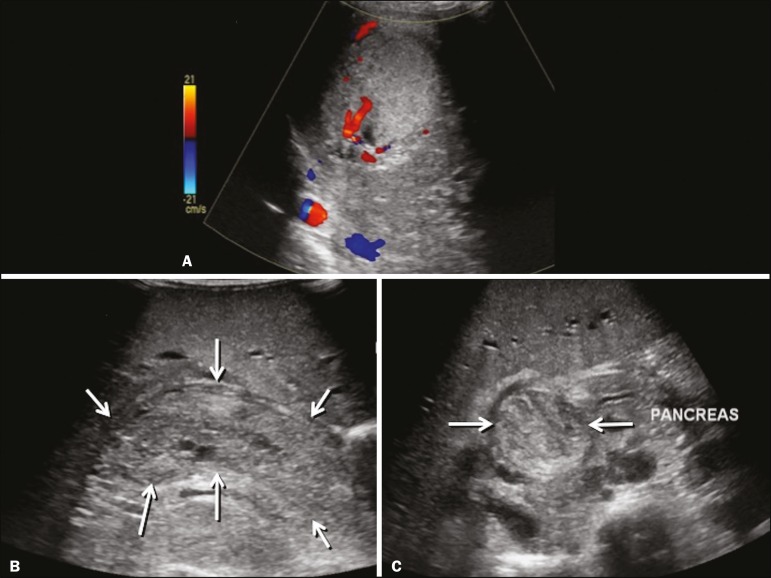
Biliary rhabdomyosarcoma in a 4-year-old girl with a three-week history of jaundice, acholic stools, abdominal pain, and pruritus, who was referred for investigation of a suspected cyst in the common bile duct. A: Doppler ultrasound of the abdomen, showing a hyperechoic solid liver lesion with central and peripheral vascularization. B,C: Lesion in the lumen of the common bile duct (longitudinal and transverse sections, respectively), occupying and significantly increasing its caliber (arrows).The lesion is solid and hyperechoic, containing cystic foci. The image in C also shows the obstructive effect produced by the lesion with repercussions for the pancreatic duct, the caliber of which is slightly increased. The mass was surgically resected, and the pathology study indicated biliary rhabdomyosarcoma with hepatic invasion.

**Presentation on ultrasound** - Dilatation of bile ducts with intraluminal mass, indicating the biliary origin of the tumor (Figure 7). Cystic areas can be identified within the mass and probably represent necrosis. Other findings include invasion of adjacent organs and regional lymphadenopathy. Although the mass may displace the portal vein, there have been no reports of portal thrombosis associated with this tumor.

## REGENERATIVE NODULE

A regenerative nodule is a non-neoplastic lesion that develops in a cirrhotic liver and is therefore also known as a cirrhotic nodule. It is characteristically surrounded by fibrous septa and may progress to a dysplastic nodule or hepatocellular carcinoma. It is distinguished from nodular regenerative hyperplasia, which develops in a liver without fibrosis and is surrounded by normal liver parenchyma^(8)^. Regenerative nodules are classified, by size, as micronodules (< 0.3 cm), macronodules (> 0.3 cm), or giant nodules (> 5.0 cm)^(9)^.

**Figure 8:**
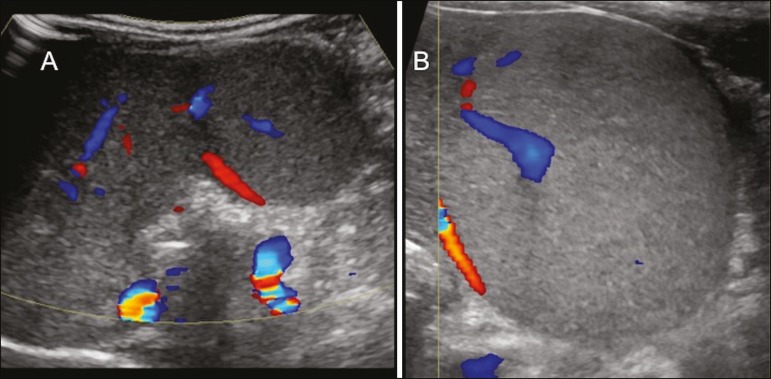
Liver regeneration nodule in a 3-month-old girl diagnosed with biliary atresia. Abdominal ultrasound with convex and linear transducers (A and B, respectively), showing a large, well-defined hyperechoic nodule in the left hepatic lobe. The image in B shows that there is no vascular distortion, and a portal branch can be seen within the lesion. Surgical resection was performed, and the pathology study indicated a regeneration nodule.

**Presentation on ultrasound** - The nodules are usually hypoechoic, but may be isoechoic, mixed, or, less frequently, hyperechoic (Figure 8)^(10)^.

## BILIARY HAMARTOMAS

Biliary hamartomas, also known as von Meyenburg complexes or microhamartomas, are rare benign malformations of the intrahepatic bile ducts and are considered part of the spectrum of fibropolycystic liver disease due to abnormal embryonic development of the ductal plaque. Biliary hamartomas are usually asymptomatic and are detected as incidental findings on imaging, in laparotomies, or during autopsies (in 6% of adults and in 1% of children).

**Figure 9:**
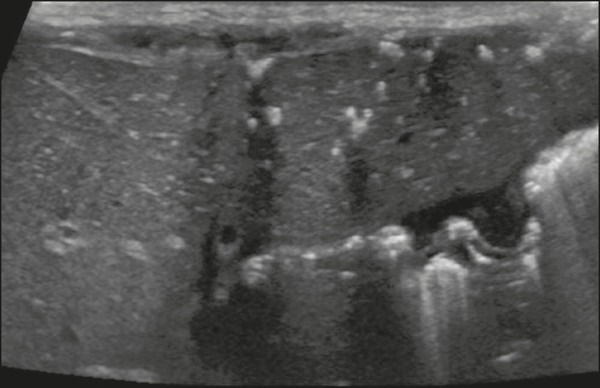
Biliary hamartomas in an otherwise healthy 2-month-old boy. Abdominal ultrasound showing multiple sparse hyperechoic foci along the biliary tract. Note that some foci produce the “comet tail” artifact, characteristic of cholesterol crystals, whereas others produce posterior acoustic shadowing, possibly corresponding to calcifications.

**Presentation on ultrasound** - Multiple small hyperechoic or hypoechoic foci with “comet tail” reverberation artifacts produced by cholesterol crystals within cystic ductal dilations or calcifications^(11)^, which can also produce posterior acoustic shadowing artifacts, as depicted in Figure 9.

## CONCLUSION

Focal hyperechoic liver lesions are commonly seen in adults, in whom hemangiomas account for most of the cases, whereas in children there is a much wider range of diagnostic possibilities for such lesions.

In pediatric patients, hyperechoic liver lesions are often identified as incidental findings in abdominal ultrasound examinations. Knowledge of the spectrum of these lesions and the imaging features that characterize them, as well as of the age group in which they are most prevalent, of their clinical manifestations, and of the accompanying biochemical alterations, as well as of any history of liver disease, trauma, or intervention, make it possible to restrict the diagnosis to the most probable hypotheses. That can inform pediatrician decision-making regarding the management of such lesions-watchful waiting or further investigation-which should always have the objective of avoiding unnecessary invasive examinations.
